# Changes in Attack Execution Following a Fencing-Specific Training Program in Competitive Male Sabre Fencers: An Exploratory Study

**DOI:** 10.3390/sports14090376

**Published:** 2026-09-01

**Authors:** Rareș Dumitrescu, Denisa-Iulia Brus, Ștefan Teriș, Răzvan-Sandu Enoiu

**Affiliations:** 1 Interdisciplinary Doctoral School, Transilvania University of Brașov, 500036 Brașov, Romania; dumitrescu_rares@yahoo.com; 2 Department of Motor Performance, Transilvania University of Brașov, 500036 Brașov, Romania; stefan.teris@unitbv.ro (Ș.T.); razvan.enoiu@unitbv.ro (R.-S.E.)

**Keywords:** sabre fencing, attack execution, fencing-specific training, technical performance, proprioception

## Abstract

Attack execution is a fundamental determinant of performance in sabre fencing, requiring athletes to integrate technical precision, tactical decision-making, perceptual abilities, and neuromuscular control within extremely short time intervals. The purpose of this exploratory single-group pre–post study was to investigate changes in attack execution, balance, postural control, and movement accuracy following an eight-week fencing-specific training program in competitive male sabre fencers. Eight competitive junior male sabre fencers completed an eight-week intervention consisting of two mesocycles designed to develop technical execution, balance, and movement control. Technical performance was assessed using four fencing-specific attack tests, whereas balance, postural control, and movement accuracy were evaluated using three sport-specific tests. Pre- and post-intervention performances were compared using paired-samples *t*-tests or exact Wilcoxon signed-rank tests according to the distribution of the paired differences, with effect sizes and 95% confidence intervals also reported. Balance in the En Garde Position increased from 44.00 ± 13.34 to 60.13 ± 18.80 s and was the only outcome showing a statistically significant pre–post change (*p* = 0.016; r_rb = 0.94). Change of Attack Direction increased from 7.50 ± 0.93 to 8.00 ± 0.93 points (*p* = 0.125; r_rb = 1.00; 4 non-tied pairs), while Beat Attack Execution increased from 8.25 ± 1.28 to 9.13 ± 0.83 points (*p* = 0.063; r_rb = 1.00; 5 non-tied pairs); neither change reached statistical significance. The remaining outcomes also showed non-significant pre–post changes. Overall, the findings indicate changes in technical and balance-related performance following the eight-week training period; however, given the absence of a control group, the small sample size, and the lack of test–retest reliability data for the sport-specific assessments, these findings should be interpreted cautiously and cannot establish a causal effect of the training program.

## 1. Introduction

Fencing is an Olympic combat sport characterized by rapid interactions between two opponents and the continuous integration of technical, tactical, physical, and psychological demands [1]. Among the three fencing weapons, sabre is characterized by particularly short action times and high movement velocities, requiring precise coordination of footwork and blade actions under substantial temporal constraints [2]. Offensive actions are central to competitive sabre performance, and their successful execution depends on technical accuracy, movement timing, distance management, and the ability to adapt the action to changing competitive situations [3,4,5]. Accordingly, the refinement of attack execution represents an important component of the long-term preparation of competitive sabre fencers.

Technical preparation in sabre fencing involves repeated practice of sport-specific actions, individualized lessons, and exercises designed to refine the coordination, accuracy, and consistency of offensive techniques [3,6,7,8]. Attacks with changes of line, beat attacks, direct attacks, and attacks directed toward specific target areas require coordinated lower- and upper-limb actions and precise blade control [7,9,10]. Biomechanical characteristics of fencing movements, including lower-limb function and lunge mechanics, may also influence the execution of sport-specific actions [10,11,12,13]. Consequently, training approaches aimed at technical development commonly combine sport-specific practice with exercises targeting the physical and neuromuscular capacities required to maintain effective movement execution.

In addition to technical execution, postural control and proprioceptive information are relevant to fencing-specific movement. Fencers must maintain and recover the en garde position while performing rapid advances, retreats, changes of direction, and lunges, requiring continuous regulation of body position and dynamic stability. Proprioceptive information contributes to the perception and control of body position and movement and, together with visual and vestibular information, is relevant to postural control and movement accuracy. This may be particularly important when visual information is reduced during sport-specific actions. Accordingly, assessments of balance, postural control, and movement accuracy under reduced visual input may provide complementary information about non-visual sensory contributions to fencing-specific movement. Such assessments should not, however, be interpreted as isolating proprioception, because performance remains dependent on the integration of multiple sensory and motor processes.

Despite the importance of technical execution and postural control in fencing, relatively few intervention studies have examined longitudinal changes in fencing-specific attack performance, particularly in competitive male sabre fencers [3,9,14,15]. Much of the existing literature has focused on physiological, biomechanical, or descriptive performance characteristics, while evidence concerning changes in sport-specific technical and balance-related outcomes over structured training periods remains limited [10,13,14,15,16]. Studies involving highly trained fencers are particularly relevant because their high baseline proficiency may restrict the magnitude of detectable changes in sport-specific performance.

Therefore, the aim of the present study was to examine pre–post changes in fencing-specific technical performance, balance, postural control, and movement accuracy following an eight-week structured training period in competitive male sabre fencers. It was hypothesized that post-intervention performance would differ from baseline in the assessed technical and balance-related outcomes. Given the single-group pre-test/post-test design, these changes were examined as within-subject differences and were not interpreted as establishing a causal effect of the training program.

## 2. Materials and Methods

### 2.1. Study Design

This study employed a single-group pre-test/post-test design to examine changes in attack execution in male sabre fencers following an eight-week structured training program. The study was designed to evaluate within-subject changes in technical performance over the training period. The intervention was conducted during the pre-competitive phase of the annual training cycle and lasted eight weeks, comprising two consecutive mesocycles organized into eight weekly microcycles. Participants completed baseline assessments before the beginning of the intervention and identical post-intervention assessments following the completion of the experimental program.

The absence of a control group reflects the practical constraints associated with conducting intervention studies in elite sport settings. All participating athletes were members of the Romanian National Olympic Training Centre and followed the same centralized preparation programme for official competitions. Consequently, allocating athletes to different training programmes or withholding the intervention from a subgroup was considered neither practically feasible nor ethically appropriate within the context of their competitive preparation. Therefore, a single-group pre-test/post-test design was selected as the most appropriate methodological approach under these real-world conditions.

All outcome measures were assessed under identical testing conditions before and after the intervention to ensure the comparability of the collected data. Furthermore, all assessments were performed by the same evaluator using identical testing procedures, equipment, and environmental conditions in order to minimize procedural variability and improve comparability between the two testing sessions.

The experimental training program was integrated into the athletes’ regular training schedule and was specifically designed to improve attack execution through progressive technical and tactical training, attack-specific drills, individualized lessons, video-assisted performance analysis, sport-specific physical preparation, psychological training, and recovery strategies. Throughout the intervention, the training load and technical complexity were progressively adjusted to facilitate the acquisition, refinement, and stabilization of attack techniques under conditions closely resembling competitive fencing.

The intervention was structured into two experimental mesocycles with distinct but complementary objectives. The first mesocycle focused primarily on technical refinement, attack modelling, and the development of sport-specific physical and tactical capacities. The second mesocycle emphasized the optimization of attack execution under competition-specific conditions while maintaining technical consistency and competitive readiness. A summary of the experimental training program is presented in Table 1, whereas a detailed weekly description of the intervention is provided in the Supplementary Materials (Tables S1 and S2).

### 2.2. Participants

Eight male sabre fencers (age: 16–20 years) participated in this study. All athletes were actively engaged in high-performance fencing and were members of the Romanian National Olympic Training Centre in Brașov. Most participants had achieved podium finishes at national and international junior competitions, indicating a high level of technical proficiency and competitive experience.

Participants were eligible for inclusion if they met the following criteria: (i) specialization in male sabre fencing; (ii) age between 16 and 20 years; (iii) active participation in the high-performance training program of the Romanian National Olympic Training Centre; and (iv) completion of both the pre-intervention and post-intervention assessments. Athletes were excluded if they missed either testing session, failed to complete the experimental training program, sustained an injury or medical condition preventing full participation, or were unable to perform the testing procedures under standardized conditions.

Before participation, all athletes received detailed information regarding the study procedures and provided written informed consent. For participants younger than 18 years, written informed consent was additionally obtained from a parent or legal guardian. All procedures were conducted in accordance with internationally accepted ethical principles for research involving human participants.

### 2.3. Experimental Training Program

The experimental training program was implemented over an eight-week period during the pre-competitive phase of the annual training cycle. The intervention consisted of two consecutive mesocycles, each comprising four weekly microcycles. Every microcycle included six training sessions followed by one recovery day, resulting in a total of 48 structured training sessions throughout the intervention.

The training program was progressively organized according to the objectives established for each mesocycle. The first mesocycle emphasized the development of sport-specific physical fitness, technical refinement, attack modelling, and the improvement of technical-tactical skills. The second mesocycle focused on optimizing attack execution under conditions closely resembling competitive fencing while maintaining technical consistency, tactical efficiency, and competitive readiness.

Training sessions integrated technical instruction, technical-tactical exercises, individualized lessons, attack-specific drills, sport-specific physical conditioning, psychological preparation, theoretical instruction, video-assisted performance analysis, and recovery strategies. Particular emphasis was placed on correcting movement biomechanics, improving proprioceptive control, and refining the technical execution of attack techniques throughout the intervention.

The eight-week training period comprised six 90 min training sessions per week, resulting in a total of 48 scheduled training sessions. The number of fencing bouts performed per athlete was progressively increased across the eight weekly microcycles, with 0, 0, 2, 2, 3, 3, 5, and 5 bouts, respectively, corresponding to a total of 20 bouts per athlete during the intervention. Individual technical lessons were incorporated throughout the program, with one lesson per athlete during the first microcycle and two lessons per athlete during each of the subsequent seven microcycles, resulting in a total of 15 individual lessons per athlete. Recovery sessions were scheduled during microcycles 4 and 8. The progressive increase in bout exposure was used to increase competition-specific demands across the training period.

### 2.4. Outcome Measures

Participants were evaluated before and after the experimental intervention using a battery of fencing-specific assessments designed to measure changes in technical performance, balance, postural control, and movement accuracy.

The assessment protocol consisted of two complementary components. The first component evaluated the technical execution of attack techniques through four fencing-specific performance tests designed to assess attack accuracy, movement coordination, timing, and technical execution. The second component assessed balance, postural control, and movement accuracy using three fencing-specific tests, two of which were performed under conditions of reduced visual input.

All tests were administered under identical experimental conditions before and after the intervention, and the same testing sequence was maintained during both evaluation sessions. Before each assessment, participants received task-specific verbal instructions describing the required movement, performance criterion, and test endpoint. The same evaluator administered all assessments at both testing sessions, and the same task-specific instructional content and testing procedures were used during the pre- and post-intervention assessments. However, a verbatim written instruction script was not included in the original testing protocol. Consequently, the exact wording used for each test, including whether specific verbal cues directed attention internally toward body position, externally toward the blade or target, holistically toward the overall movement, or toward a core component of the task, cannot be reliably reconstructed retrospectively. No attempt was therefore made to retrospectively assign an attentional-focus category to the original instructions. Blinded video-based scoring was not performed. The outcome measures used in the study are described in the following subsections.

#### 2.4.1. Technical Performance Assessment

Technical performance was evaluated using four fencing-specific tests designed to assess the execution of fundamental attack techniques in male sabre fencing. Each test consisted of ten repetitions, and only correctly executed actions were recorded as successful attempts. All assessments were conducted individually under standardized conditions by the same evaluator, who provided identical stimuli, instructions, and evaluation criteria throughout the testing sessions.

The stimulus in each technical assessment was provided by the evaluator through a predetermined fencing-specific blade or arm action rather than by a verbal command. In the Arm Control during Forward Movement test, the stimulus consisted of a change in the direction of the evaluator’s blade while the athlete advanced. In the Change of Attack Direction and Beat Attack Execution tests, the stimulus was delivered during the final phase of the attack through closure of the attack line or interposition of the evaluator’s blade, respectively. In the Attack to the Forearm test, the stimulus consisted of extension of the evaluator’s weapon arm to expose the valid forearm target. The same stimulus procedures and success criteria were applied during the pre- and post-intervention assessments.

##### Arm Control During Forward Movement

This test evaluated the athlete’s ability to control blade position while advancing. The athlete moved forward at a moderate speed toward the coach, who provided a visual stimulus by changing the direction of the blade while remaining in a defensive position. Upon receiving the stimulus, the athlete was required to direct the tip of the sabre toward the opposite line of the coach’s blade. Attempts in which the athlete failed to respond, responded before the stimulus, or reacted without an appropriate stimulus were recorded as unsuccessful.

##### Change of Attack Direction

This test assessed the athlete’s ability to modify the direction of the attack during its final phase. As the athlete initiated the final phase of the attack, the coach closed the line of attack by positioning the blade in front of the athlete’s weapon. The athlete was required to immediately redirect the attack toward the newly opened target. Attempts in which the athlete changed the direction before the coach’s stimulus or without an appropriate stimulus were recorded as unsuccessful.

##### Beat Attack Execution

This test evaluated the execution of a beat attack in response to an opponent’s defensive blade position. During the final phase of the attack, the coach intentionally placed the blade between the athlete’s weapon and the target. The athlete was required to perform a blade beat to remove the opponent’s blade and immediately complete the attack through the newly created opening. Attempts in which the athlete failed to execute the blade beat when required or unnecessarily searched for the opponent’s blade were recorded as unsuccessful.

##### Attack to the Forearm

This test assessed the athlete’s ability to recognize and exploit an opening to the opponent’s forearm. While assuming a defensive position, the coach extended the weapon arm to expose the valid target area of the forearm. The athlete was required to complete the attack by accurately striking the exposed target. Ten repetitions were performed, consisting of five attacks directed to the inner side of the forearm and five attacks directed to the outer side. Only correctly executed hits on the valid target area were recorded as successful.

#### 2.4.2. Assessment Under Reduced Visual Input and Balance Conditions

Balance, postural control, and movement accuracy were assessed using three fencing-specific tests. Two of these assessments were performed under visual occlusion. Removal of visual input was used to alter the sensory and attentional conditions of the tasks and to increase reliance on non-visual information, including proprioceptive and vestibular cues. Accordingly, these assessments were not considered to isolate proprioception, but rather to evaluate fencing-specific performance under conditions of reduced visual input. All assessments were conducted under standardized conditions before and after the intervention by the same evaluator using identical testing procedures and equipment.

##### Balance in the En Garde Position

Balance was assessed while the athlete maintained the en garde position on two balance hemispheres, with the front foot positioned on one hemisphere and the rear foot on the other. Timing commenced once the athlete achieved a stable position without movement. The outcome measure was the duration (seconds) for which the athlete was able to maintain balance without losing the correct fencing posture. Timing began once the athlete had stabilized in the required en garde position. No predefined maximum duration was imposed; the test ended when the athlete was no longer able to maintain the required stable position.

##### Maintenance of the Correct Fencing Position

This test evaluated the athlete’s ability to maintain the correct fencing posture under conditions of visual occlusion. While blindfolded, the athlete performed ten attacks consisting of a two-step advance followed by a lunge toward a fencing dummy. After each attack, the athlete returned to the starting position using two backward steps and re-established the en garde position. Only attacks that successfully struck the valid target area of the dummy were recorded as successful. A successful attempt was defined as an attack that contacted the valid target area of the mannequin.

##### Straight-Line Movement Test

This test assessed the athlete’s ability to maintain a straight movement trajectory under conditions of visual occlusion. While blindfolded, the athlete advanced along the fencing piste in the en garde position using standard fencing footwork. Performance was evaluated by measuring the lateral deviation from a reference line marked along the piste. Athletes were instructed to perform continuous forward movement without interruptions or pauses between steps. Performance was quantified as the lateral deviation, expressed in centimetres, from a reference line marked longitudinally along the fencing piste. Lower values indicated better maintenance of the intended movement trajectory and therefore better performance.

### 2.5. Statistical Analysis

Data were analysed using IBM SPSS Statistics version 29.0 (IBM Corp., Armonk, NY, USA). Descriptive statistics are presented as mean ± standard deviation (SD). For pre–post comparisons, the normality of the individual difference scores (post-intervention minus pre-intervention) was assessed using the Shapiro–Wilk test. When the difference scores did not significantly deviate from normality, paired-samples *t*-tests were applied. When the normality assumption was violated, exact two-tailed Wilcoxon signed-rank tests were used. Statistical significance was set at *p* < 0.05. For paired-samples *t*-tests, effect sizes were calculated as Cohen’s dz, defined as the mean paired difference divided by the standard deviation of the paired differences. For Wilcoxon signed-rank tests, effect sizes were expressed as matched-pairs rank-biserial correlations (r_rb). For Wilcoxon signed-rank analyses, the number of non-tied pairs (i.e., participants with a non-zero pre–post difference) was also reported alongside r_rb, because tied observations do not contribute to the signed-rank effect-size estimate. Given the small sample size, effect sizes and confidence intervals were considered alongside *p*-values when interpreting the findings.

Ninety-five percent confidence intervals (95% CIs) for mean pre–post changes were estimated using bias-corrected and accelerated (BCa) bootstrap resampling with 10,000 resamples.

### 2.6. Ethical Considerations

The study was conducted in accordance with the ethical principles of the Declaration of Helsinki. Ethical approval for the study was obtained from the Research Ethics Commission of the Faculty of Physical Education and Mountain Sports, Transilvania University of Brașov, Romania. The research protocol entitled “Effects of a Fencing-Specific Training Program on Attack Execution in Competitive Male Sabre Fencers” was reviewed and approved by the Commission prior to the commencement of the study (Approval No. 177/29.07.2026). All participants provided written informed consent before enrolment in the study. For participants under 18 years of age, written informed consent was additionally obtained from their parents or legal guardians. All data were anonymized prior to analysis to ensure participant confidentiality.

## 3. Results

### 3.1. Technical Performance

Changes in technical performance following the eight-week experimental training program were evaluated using four fencing-specific tests assessing attack execution in male sabre fencers. The results of the pre- and post-intervention assessments are summarized in Table 2. Overall, mean performance improved across all technical tests after the intervention, although the magnitude and statistical significance of these changes varied among the evaluated attack techniques.

#### 3.1.1. Arm Control During Forward Movement

Mean performance in the Arm Control during Forward Movement test increased from 8.88 ± 1.13 at baseline to 9.50 ± 0.53 following the intervention, corresponding to a mean change of +0.63 points. This change did not reach statistical significance (*p* = 0.140), with a moderate effect size (Cohen’s dz = 0.59).

#### 3.1.2. Change of Attack Direction

Mean performance in the Change of Attack Direction test increased from 7.50 ± 0.93 at baseline to 8.00 ± 0.93 following the intervention, corresponding to a mean change of +0.50 points (95% CI: −0.13 to 0.88). However, the change did not reach statistical significance according to the exact Wilcoxon signed-rank test (*p* = 0.125), despite a large matched-pairs rank-biserial effect size (r_rb = 1.00), based on four non-tied pairs. Four participants showed a positive pre–post change, while the remaining four showed no change. Thus, the r_rb value of 1.00 indicates that all non-tied differences were in the direction of improvement and should not be interpreted as a maximal effect across all eight participants.

#### 3.1.3. Beat Attack Execution

Mean performance in the Beat Attack Execution test increased from 8.25 ± 1.28 at baseline to 9.13 ± 0.83 following the intervention, corresponding to a mean change of +0.88 points (95% CI: 0.38 to 1.75). The change did not reach the conventional threshold for statistical significance according to the exact Wilcoxon signed-rank test (*p* = 0.063), although the matched-pairs rank-biserial effect size was large (r_rb = 1.00), based on five non-tied pairs. Five participants showed a positive pre–post change, while three showed no change. Thus, the r_rb value of 1.00 indicates that all non-tied differences were in the direction of improvement rather than a maximal effect across all eight participants.

#### 3.1.4. Attack to the Forearm

Mean performance in the Attack to the Forearm test increased from 8.63 ± 1.19 at baseline to 9.13 ± 0.83 following the intervention, corresponding to a mean change of +0.50 points. This change was not statistically significant (*p* = 0.227), with a moderate effect size (Cohen’s dz = 0.47).

### 3.2. Balance and Performance Under Reduced Visual Input

Changes in balance, postural control, and movement accuracy following the eight-week experimental training program were evaluated using three fencing-specific tests. The results of the pre- and post-intervention assessments are summarized in Table 3. Numerical changes were observed across all three outcomes, although statistical significance was reached only for Balance in the En Garde Position.

#### 3.2.1. Balance in the En Garde Position

Balance in the En Garde Position increased from 44.00 ± 13.34 s at baseline to 60.13 ± 18.80 s following the intervention, corresponding to a mean change of +16.13 s (95% CI: 4.97 to 21.13). The change was statistically significant according to the exact Wilcoxon signed-rank test (*p* = 0.016), with a large matched-pairs rank-biserial effect size (r_rb = 0.94; eight non-tied pairs).

#### 3.2.2. Maintenance of the Correct Fencing Position

Mean performance in the Maintenance of the Correct Fencing Position test increased from 7.88 ± 2.30 to 8.88 ± 1.25 successful attempts, corresponding to a mean change of +1.00 successful attempt. The change did not reach statistical significance according to the exact Wilcoxon signed-rank test (*p* = 0.188), although the matched-pairs rank-biserial effect size was large (r_rb = 0.71; six non-tied pairs). Two participants showed no pre–post change.

#### 3.2.3. Straight-Line Movement Test

Performance in the Straight-Line Movement Test showed a reduction in mean deviation from the reference line, from 33.88 ± 12.98 cm at baseline to 30.75 ± 9.74 cm following the intervention, corresponding to a mean change of −3.13 cm. The change did not reach statistical significance (*p* = 0.115), with a moderate effect size (Cohen’s dz = −0.64). Lower deviation values represent better performance in this test.

## 4. Discussion

The present study examined pre–post changes in fencing-specific technical performance, balance, postural control, and movement accuracy following an eight-week structured training period in competitive male sabre fencers. Balance in the En Garde Position was the only outcome showing a statistically significant pre–post change. Change in Attack Direction and Beat Attack Execution showed numerical increases but did not reach statistical significance, while Arm Control during Forward Movement, Attack to the Forearm, Maintenance of the Correct Fencing Position, and Straight-Line Movement also showed non-significant pre–post changes. Given the single-group design, small sample size, and absence of test–retest reliability data for the sport-specific assessments, these findings should be interpreted as exploratory within-subject changes rather than evidence of the effectiveness of the training program.

To better understand the practical significance of these findings, they should be interpreted within the context of the specific performance demands of modern sabre fencing. During offensive exchanges, successful attack execution depends on the athlete’s ability to continuously adapt blade trajectory, movement timing, and interpersonal distance in response to the opponent’s actions. The improvements observed in the present study are consistent with these sport-specific demands, where successful attack execution relies on the effective integration of technical precision, movement speed, tactical decision-making, and perceptual-cognitive abilities. Unlike the other fencing weapons, sabre is characterized by extremely short action times, requiring athletes to perceive external stimuli, rapidly select the appropriate offensive response, and execute the attack within fractions of a second [2]. These characteristics provide relevant context for interpreting the fencing-specific technical outcomes assessed in the present study. However, perceptual processing, tactical decision-making, and competitive performance were not directly measured and therefore cannot be inferred from the present results.

Among the technical outcomes, the largest numerical increases were observed in the Change of Attack Direction and Beat Attack Execution tests, although neither change reached statistical significance. Both offensive actions require athletes to continuously adapt blade trajectory and movement timing according to the opponent’s behaviour while maintaining technical accuracy throughout the attack. These findings are consistent with those reported by Witkowski [3], who emphasized that the effectiveness of offensive actions in elite sabre fencing depends not only on movement speed but also on the quality, precision, and consistency of technical execution under rapidly changing competitive conditions. Likewise, Varesco [9] demonstrated that successful fencing performance is closely associated with the interaction between attack speed, movement accuracy, and attentional processes, highlighting that technical precision and perceptual abilities contribute independently to successful offensive performance.

The absence of statistically significant changes in Arm Control during Forward Movement and Attack to the Forearm should also be considered in relation to the high baseline performance of the participants. Mean pre-intervention scores were 8.88 and 8.63 out of a maximum of 10 points, respectively, leaving relatively limited room for further measurable improvement. This restricted score range may have introduced a ceiling effect, reducing the sensitivity of these tests to detect relatively small changes in already highly proficient fencers. Therefore, the non-significant findings for these technical outcomes may reflect, at least in part, limitations in measurement sensitivity at the upper end of the scoring scale rather than an absence of change.

The observed numerical changes occurred during a training period characterized by a high degree of fencing specificity. Offensive actions such as changing the attack line and executing beat attacks require accurate coordination between lower-limb footwork, upper-limb blade control, and distance regulation. According to Czajkowski [6] and Forteza de la Rosa [7], repeated sport-specific practice represents an important component of technical preparation in fencing. However, because the present study did not include a control group, the observed changes cannot be attributed specifically to the training content or its degree of specificity.

From a theoretical perspective, perception–action frameworks emphasize the interaction between sensory information, decision-making, and motor execution during complex sport-specific actions. Such processes are likely to be relevant to competitive sabre fencing, in which athletes respond to rapidly changing opponent behaviour and interpersonal distance. However, perceptual processing, anticipation, tactical decision-making, and perception–action coupling were not directly assessed in the present study. Consequently, their potential contribution to the observed pre–post changes remains speculative and should not be interpreted as a mechanism demonstrated by the present findings.

The observed pre–post changes occurred during a training period characterized by a high degree of fencing specificity and the integration of multiple performance components. Rather than focusing exclusively on isolated technical repetition, the intervention combined individualized coaching, fencing-specific offensive drills, proprioceptive exercises, and video-assisted feedback, thereby reproducing many of the perceptual, temporal, and tactical demands encountered during competitive bouts. Such an approach is consistent with the principle of training specificity, which states that motor adaptations are optimized when practice closely resembles the movement patterns, environmental constraints, and decision-making requirements encountered during competition [3,6,7,12].

From a motor-learning perspective, representative practice has been proposed to facilitate the acquisition and refinement of sport-specific movement patterns [17,18]. Although the training program used in the present study incorporated fencing-specific situations intended to reproduce elements of competitive practice, the present assessments evaluated performance outcomes rather than the perceptual or cognitive processes underlying their execution. Therefore, no conclusions can be drawn regarding changes in perception–action coupling, anticipation, or decision-making [2,9,12,17].

Video-assisted feedback represented one component of the training program. Previous research has suggested that systematic movement analysis combined with structured technical feedback may facilitate the identification and correction of movement deficiencies [19]. However, because the contribution of individual training components was not assessed separately in the present study, no specific effect can be attributed to video-assisted feedback. Observational learning enables athletes to compare their own movement execution with an optimal technical model, facilitating error detection, self-evaluation, and movement correction. When integrated with immediate coach feedback, video analysis may accelerate technical refinement, improve movement consistency, and promote more efficient motor learning. Recent studies have shown that augmented feedback and systematic video analysis enhance technical performance, movement accuracy, and decision-making in athletes performing complex sport-specific skills [9,14,20].

In addition to the technical outcomes, pre–post changes were observed in balance, postural control, and movement accuracy. The most pronounced change occurred in the Balance in the En Garde Position test, which was the only outcome to reach statistical significance. Maintenance of the Correct Fencing Position and Straight-Line Movement showed numerical changes but did not reach statistical significance. These findings should be interpreted cautiously, particularly because the assessments performed under visual occlusion do not isolate proprioception and because the balance result may have been influenced by familiarisation and test–retest learning.

An additional methodological consideration concerns the potential role of attentional focus during the assessments. Instructional wording can influence how athletes allocate attention during motor-task execution, and different attentional-focus strategies, including internal, external, holistic, and core-component foci, may affect performance even in highly trained athletes [21,22]. In the present study, the exact verbal instructions cannot be reliably reconstructed because a verbatim instruction script was not prospectively documented. Furthermore, the nature of the tasks may have directed attention differently: the technical assessments involved blade- or target-related information, whereas visual occlusion during the balance and movement-control assessments may have altered the sensory information available and the athletes’ attentional priorities. Accordingly, attentional focus should be regarded as a potential uncontrolled factor rather than as a mechanism assessed by the present study.

Balance and proprioceptive control play a fundamental role in sabre fencing because athletes are required to maintain postural stability while performing rapid accelerations, decelerations, changes of direction, and offensive actions under continuously changing competitive conditions. During attack execution, the fencer must generate high movement velocity while simultaneously preserving body alignment and precise blade control. Any loss of postural stability may compromise movement efficiency, reduce blade accuracy, and ultimately decrease the probability of successfully scoring a touch. Consequently, proprioceptive abilities should not be considered independent physical capacities but rather integral components of technical performance that support the execution of complex offensive actions [2,3,12]. From a biomechanical perspective, maintaining dynamic stability during the lunge may facilitate efficient force transmission from the lower limbs through the trunk to the weapon arm and may contribute to movement efficiency and attack accuracy while limiting unnecessary compensatory movements.

However, the significant pre–post change observed in the Balance in the En Garde Position test should be interpreted cautiously. No separate familiarisation session was conducted before baseline testing, the task did not include a predefined maximum duration, and the evaluator was not blinded to the testing occasion. Therefore, part of the observed increase in performance may reflect greater familiarity with the testing procedure, improved task-specific strategy, or other test–retest learning effects rather than a training-related change. Given these methodological limitations, the present study cannot determine the extent to which the observed improvement in balance was attributable to the training program itself.

The present findings are consistent with previous research emphasizing the importance of proprioceptive training for improving neuromuscular function and movement quality in fencing athletes. De Vasconcelos [23,24] reported that proprioceptive training enhances dynamic balance, postural control, and movement efficiency by improving the coordination of neuromuscular responses during sport-specific tasks. Similarly, Varesco [9] highlighted that successful fencing performance depends not only on technical proficiency but also on the athlete’s ability to maintain movement stability while executing rapid offensive and defensive actions. The observed balance change is consistent with previous research reporting associations between proprioceptive training and balance-related performance in fencers [23,24]; however, the present design does not allow this change to be attributed specifically to the proprioceptive components of the training program.

The absence of statistically significant improvements in the remaining proprioceptive variables should be interpreted with caution. The participating athletes were competitive junior sabre fencers with a relatively high initial level of technical proficiency, which may have limited the magnitude of measurable improvements over an eight-week intervention. Furthermore, maintaining the correct fencing position and linear movement with visual occlusion represent highly complex motor tasks requiring continuous integration of proprioceptive, vestibular, and motor control processes. It is therefore plausible that longer intervention periods or greater training volumes would be required to produce statistically significant changes in these variables.

From a practical perspective, the training period examined in the present study illustrates an integrated approach combining technical, tactical, balance-related, and perceptual components within a fencing-specific training environment. Offensive actions such as changing the attack direction and executing beat attacks were practiced under conditions involving external stimuli, distance regulation, and adaptation of technical responses. Although the present design does not allow conclusions regarding the effectiveness of these specific training components, such practice conditions are consistent with the sport-specific demands described in the fencing literature [3,9].

The present findings also emphasize the value of individualized coaching as an essential component of technical development in sabre fencing. Individual lessons allow coaches to provide immediate feedback regarding blade trajectory, movement timing, body alignment, and tactical decision-making while adapting training content to the specific needs of each athlete. When combined with representative practice situations, individualized instruction may accelerate the refinement of complex offensive actions and improve the consistency of technical execution under competitive conditions [6,7].

Another component of the training program was the inclusion of balance-related exercises and video-assisted feedback within the regular training process. These components may be relevant to postural control and technical development; however, their individual contributions to the observed changes cannot be determined from the present study design. Similarly, video analysis may provide athletes and coaches with information regarding technical execution and movement patterns, although its specific effect was not evaluated independently in the present study [9,14,20].

Taken together, the present findings provide preliminary descriptive information regarding technical and balance-related changes occurring during an integrated period of sabre fencing training. Although the training program combined technical instruction, representative practice, balance-related exercises, and systematic performance analysis, the present design does not allow the contribution of these individual components to be determined. Controlled studies are required before specific recommendations regarding the effectiveness of this integrated approach can be made.

### Limitations

Several limitations should be considered when interpreting the present findings. First, the small sample size (n = 8) and the single-group pre-test/post-test design limit both the generalizability of the results and the ability to attribute the observed changes to the training program. Without a control condition, changes occurring over the eight-week period cannot be separated from normal training progression, seasonal development, competitive experience, maturation, or other uncontrolled factors. The findings should therefore be interpreted as within-subject pre–post changes rather than evidence of a causal effect of the intervention.

Second, test–retest reliability and measurement error were not established for the fencing-specific outcome measures. A separate reliability protocol was not conducted; therefore, intraclass correlation coefficients (ICC), typical error (TE), and minimal detectable change (MDC) could not be determined. Consequently, particularly for the relatively small absolute changes observed in several technical outcomes, it cannot be established whether the observed differences exceeded the measurement error of the tests. Future studies should establish the reliability and measurement properties of these assessments before using them to quantify longitudinal changes. In addition, some procedural details relevant to full replication of the sport-specific assessments, including the exact athlete–evaluator distance, the dimensions of the balance hemispheres, and the precise reference point and measuring instrument used to quantify lateral deviation, were not documented in sufficient detail in the original testing protocol. This limits the reproducibility of these assessments and should be addressed in future studies through fully standardized and prospectively documented testing procedures.

Third, the high baseline scores observed in some technical assessments may have produced ceiling effects. In particular, baseline scores of 8.88/10 for Arm Control during Forward Movement and 8.63/10 for Attack to the Forearm left limited room for further measurable change and may have reduced the sensitivity of the 0–10 scoring scale in these highly proficient athletes.

Fourth, potential familiarisation and evaluator-related effects should be acknowledged. No separate familiarisation session was conducted before baseline testing, and the Balance in the En Garde Position test had no predefined time cap. In addition, the evaluator was not blinded to the testing occasion. A further limitation concerns the verbal instructions provided during the sport-specific assessments. Although the same evaluator administered the assessments and the same task-specific instructional content was used at both testing sessions, a verbatim written instruction script was not included in the original protocol and the exact wording cannot be reliably reconstructed retrospectively. Consequently, it is not possible to determine whether the instructions directed athletes’ attention internally toward body position, externally toward the blade or target, holistically toward the overall movement, or toward a core action component of the task. This is relevant because instructional wording and the resulting attentional focus can influence motor performance, including in highly trained athletes [21,22]. Moreover, the different task structures may themselves have encouraged different attentional priorities; for example, the technical tests involved blade- or target-related information, whereas the tests performed with visual occlusion may have increased reliance on body-related sensory information. Therefore, differences in attentional focus represent an uncontrolled measurement factor and should be considered when interpreting the observed pre–post changes. Future studies should use standardized, verbatim instructions and report them in sufficient detail to permit replication and classification of attentional focus.

Finally, seven outcome measures were examined using multiple statistical comparisons without adjustment for multiplicity, increasing the probability of a Type I error. Given the small sample and exploratory nature of the study, the individual *p*-values should therefore be interpreted cautiously rather than as confirmatory evidence. Future research should prospectively define primary and secondary outcomes and use appropriately powered controlled designs and statistical procedures that account for multiple testing where appropriate.

## 5. Conclusions

The present study identified pre–post changes in fencing-specific technical and balance-related performance following an eight-week training period in competitive male sabre fencers. Balance in the En Garde Position was the only outcome showing a statistically significant pre–post change, whereas the changes observed in the technical and other balance-related outcomes did not reach statistical significance. These findings describe the performance changes observed over the study period but should not be interpreted as demonstrating a causal effect of the training program.

From a practical perspective, the findings highlight the importance of evaluating fencing-specific performance using multiple complementary outcomes while interpreting relatively small pre–post changes cautiously in highly trained athletes. In particular, the significant change observed in balance should be considered in light of the absence of a separate familiarisation session and the possibility of test–retest learning effects. Similarly, the high baseline scores observed in some technical assessments may have limited the ability of the 0–10 scoring scale to detect further changes. Therefore, the present results should be regarded as exploratory rather than as evidence of training effectiveness.

Future studies evaluating this training approach should employ larger samples and adequately powered controlled or randomized designs, together with reliable and validated outcome measures, standardized familiarisation and testing procedures, blinded assessment where feasible, and prospectively defined primary outcomes. Such studies are needed to determine whether changes observed following fencing-specific training exceed measurement and learning effects and can be attributed to the training program itself.

## Figures and Tables

**Table 1:** Summary of the experimental training program.

Experimental Phase	Duration	Primary Objectives	Main Training Contents
Mesocycle 1 (Microcycles 1–4)	4 weeks	Development of sport-specific fitness; technical refinement; attack modelling; improvement of tactical and psychological abilities	Technical training; technical-tactical training; attack modelling; individual lessons; sport-specific physical preparation; psychological preparation; video analysis; theoretical instruction; recovery; baseline assessment
Mesocycle 2 (Microcycles 5–8)	4 weeks	Optimization of attack execution; technical-tactical refinement; competition-specific preparation; maintenance of performance	Technical training; technical-tactical training; competition-specific attack drills; individual lessons; sport-specific physical preparation; video analysis; theoretical instruction; recovery; post-intervention assessment

**Table 2:** Changes in technical performance following the eight-week experimental training program.

Statistic	Arm Control During Forward Movement	Change of Attack Direction	Beat Attack Execution	Attack to the Forearm
Pre-intervention (Mean ± SD)	8.88 ± 1.13	7.50 ± 0.93	8.25 ± 1.28	8.63 ± 1.19
Post-intervention (Mean ± SD)	9.50 ± 0.53	8.00 ± 0.93	9.13 ± 0.83	9.13 ± 0.83
Mean	+0.63	+0.50	+0.88	+0.50
95% CI	−0.13 to 1.38	−0.13 to 0.88	0.38 to 1.75	−0.25 to 1.13
Statistical test	Paired *t*-test	Exact Wilcoxon	Exact Wilcoxon	Paired *t*-test
*p*-value	0.140	0.125	0.063	0.227
Effect size	dz = 0.59	r_rb = 1.00 (4 non-tied pairs)	r_rb = 1.00 (5 non-tied pairs)	dz = 0.47

Abbreviations: SD = standard deviation; CI = confidence interval; dz = Cohen’s dz for paired-samples *t*-tests; r_rb = matched-pairs rank-biserial correlation for Wilcoxon signed-rank tests. Changes were calculated as post-intervention minus pre-intervention values. The 95% confidence intervals for mean changes were estimated using bias-corrected and accelerated (BCa) bootstrap resampling with 10,000 resamples. Statistical significance was set at *p* < 0.05.

**Table 3:** Changes in balance, postural control, and movement accuracy following the eight-week experimental training program.

Statistic	Balance in the En Garde Position	Maintenance of the Correct Fencing Position	Straight-Line Movement Test
Pre-intervention (Mean ± SD)	44.00 ± 13.34	7.88 ± 2.30	33.88 ± 12.98
Post-intervention (Mean ± SD)	60.13 ± 18.80	8.88 ± 1.25	30.75 ± 9.74
Mean	+16.13	+1.00	−3.13
95% CI	4.97 to 21.13	0.25 to 2.75	−6.50 to −0.13
Statistical test	Exact Wilcoxon	Exact Wilcoxon	Paired *t*-test
*p*-value	0.016	0.188	0.115
Effect size	r_rb = 0.94 (8 non-tied pairs)	r_rb = 0.71 (6 non-tied pairs)	dz = −0.64

Abbreviations: SD = standard deviation; CI = confidence interval; dz = Cohen’s dz for paired-samples *t*-tests; r_rb = matched-pairs rank-biserial correlation for Wilcoxon signed-rank tests. Changes were calculated as post-intervention minus pre-intervention values. The 95% confidence intervals for mean changes were estimated using bias-corrected and accelerated (BCa) bootstrap resampling with 10,000 resamples. Statistical significance was set at *p* < 0.05. For the Straight-Line Movement test, lower values indicate better performance; therefore, a negative change reflects improvement.

## Data Availability

The data that support the findings of this study are available from the corresponding author upon reasonable request.

## Supplementary Materials

The following supporting information can be downloaded at: https://www.mdpi.com/article/10.3390/sports14090376/s1, Table S1: Experimental Mesocycle 1; Table S2: Experimental Mesocycle 2; Table S3: Individual raw pre- and post-intervention data for all study outcomes.
