# Supplementary material for: Changes in Attack Execution Following a Fencing-Specific Training Program in Competitive Male Sabre Fencers: An Exploratory Study
Source: Sports (Basel). 2026 Sep 1;14(9):376. doi: 10.3390/sports14090376 (PMC13611738; doi:10.3390/sports14090376)
Supplement: Supplementary file 1 [file sports-14-00376-s001.zip › sports-4482454-supplementary.pdf]

Tabel S1. Experimental Mesocycle 1

Stages	Stage 1	Stage 2	Stage 3	Stage 4
**Period**	**28.04 – 04.05**	**05.05 –11.05 12.11**	**12.05-18.05**	**19.05 - 25.05**
**Nr of days**	7	7	7	7
**Training days**	6	6	6	6
**Rest days**	1	1	1	1
**Training sessions**	6	6	6	6
**Bouts per training session per athlete**	0	0	2	2
**Individual lessons per athlete**	1	2	2	2
**Recovery sessions**	0	0	0	1
**Competitions**	0	0	0	0
**Training phase**	Pre-competitive	Pre-competitive	Pre-competitive	Pre-competitive
**Objective**	Testing<br>Development of sport-specific work capacity<br>Technical-tactical improvement	Achievement and maintenance of peak performance<br>Improvement of attack strategy and tactics<br>Development of psychological skills	Achievement and maintenance of peak performance<br>Development of sport-specific work capacity<br>Technical-tactical improvement	Technical-tactical improvement<br>Achievement and maintenance of peak performance<br>Development of psychological skills
**Training content**	Technical training (technical refinement, attack modeling, evaluation)<br>Sport-specific physical training<br>Psychological training<br>Tactical and technical-tactical training<br>Video analysis<br>Theoretical preparation<br>Recovery<br>Testing	Attack modeling<br>Technical-tactical training<br>Theoretical preparation (competition strategy and competition analysis)<br>Video analysis<br>Recovery	Technical training (technical refinement, attack modeling, evaluation)<br>Sport-specific physical training<br>Psychological training<br>Tactical and technical-tactical training<br>Video analysis<br>Theoretical preparation	Attack modeling<br>Technical-tactical training<br>Theoretical preparation (competition strategy and competition analysis)<br>Video analysis<br>Recovery
**Training venue**	Brașov	Brașov	Brașov	Brașov

Tabel S2. Experimental Mesocycle 2

Stages	Stage 5	Stage 6	Stage 7	Stage 8
**Period**	**26.05 – 01.06**	**02.06 –08.06 12.11**	**09.06-15.06**	**16.06 - 22.06**
**Nr of days**	7	7	7	7
**Training days**	6	6	6	6
**Rest days**	1	1	1	1
**Training sessions**	6	6	6	6
**Bouts per training session per athlete**	3	3	5	5
**Individual lessons per athlete**	2	2	2	2
**Recovery sessions**	0	0	0	1
**Competitions**	0	0	0	0
**Training phase**	Pre-competitive	Pre-competitive	Pre-competitive	Pre-competitive
**Objective**	Achievement and maintenance of peak performance<br>Development of sport-specific work capacity<br>Technical-tactical improvement	Achievement and maintenance of peak performance<br>Improvement of attack strategy and tactics<br>Development of psychological skills	Achievement and maintenance of peak performance<br>Development of sport-specific work capacity<br>Technical-tactical improvement	Technical-tactical improvement<br>Achievement and maintenance of peak performance<br>Development of psychological skills<br>Testing
**Training content**	Attack modeling<br>Technical training (technical refinement, attack modeling, evaluation)<br>Sport-specific physical training<br>Psychological training<br>Tactical and technical-tactical training<br>Video analysis<br>Theoretical preparation<br>Recovery	Attack modeling<br>Technical-tactical training<br>Theoretical preparation (competition strategy and competition analysis)<br>Video analysis<br>Recovery	Attack modeling<br>Technical training (technical refinement, attack modeling, evaluation)<br>Sport-specific physical training<br>Psychological training<br>Tactical and technical-tactical training<br>Video analysis<br>Theoretical preparation.	Attack simulation<br>Technical-tactical training<br>Theoretical preparation (competition strategy and competition analysis)<br>Recovery<br>Testing
**Training venue**	Brașov	Brașov	Brașov	Brașov

Tabel S3. Individual raw pre- and post-intervention data for all study outcomes

Outcome	S1	S2	S3	S4	S5	S6	S7	S8
**Arm Control Pre**	9	10	8	10	9	7	8	10
**Arm Control Post**	9	9	9	10	10	9	10	10
**Change direction pre**	7	7	7	8	8	6	8	9
**Change direction post**	8	7	8	8	8	7	8	10
**Beat attack pre**	8	7	6	9	9	8	9	10
**Beat attack post**	9	8	9	10	10	8	9	10
**Forearm pre**	9	10	9	10	8	7	7	9
**Forearm post**	10	10	8	9	9	8	9	10
**Balance pre (s)**	45	32	55	45	56	17	49	53
**Balance post (s)**	36	54	78	58	78	32	65	80
**Correct position pre**	7	8	10	9	3	7	9	10
**Correct position post**	8	7	10	10	8	8	10	10
**Straight-line pre (cm)**	40	26	11	54	32	45	34	29
**Straight-line post (cm)**	32	21	15	43	30	42	36	27

Abbreviations: Pre = pre-intervention assessment; Post = post-intervention assessment. Arm Control, Change Direction, Beat Attack, Forearm, and Correct Position are expressed as the number of successful attempts out of 10. Balance is expressed in seconds (s). Straight-Line performance is expressed as lateral deviation in centimeters (cm), with lower values indicating better performance.
